# Association Between Maternal Caffeine Consumption and Metabolism and Neonatal Anthropometry: A Secondary Analysis of the NICHD Fetal Growth Studies–Singletons

**DOI:** 10.1001/jamanetworkopen.2021.3238

**Published:** 2021-03-25

**Authors:** Jessica L. Gleason, Fasil Tekola-Ayele, Rajeshwari Sundaram, Stefanie N. Hinkle, Yassaman Vafai, Germaine M. Buck Louis, Nicole Gerlanc, Melissa Amyx, Alaina M. Bever, Melissa M. Smarr, Morgan Robinson, Kurunthachalam Kannan, Katherine L. Grantz

**Affiliations:** 1 Epidemiology Branch, Division of Intramural Population Health Research, *Eunice Kennedy Shriver* National Institute of Child Health and Human Development, National Institutes of Health, Bethesda, Maryland; 2 Biostatistics Branch, Division of Intramural Population Health Research, *Eunice Kennedy Shriver* National Institute of Child Health and Human Development, National Institutes of Health, Bethesda, Maryland; 3 Office of the Dean, College of Health and Human Services, George Mason University, Fairfax, Virginia; 4 The Prospective Group Inc, Fairfax, Virginia; 5 Department of Pediatrics, New York University School of Medicine, New York; 6 Department of Environmental Medicine, New York University School of Medicine, New York

## Abstract

**Importance:**

Higher caffeine consumption during pregnancy has been associated with lower birth weight. However, associations of caffeine consumption, based on both plasma concentrations of caffeine and its metabolites, and self-reported caffeinated beverage intake, with multiple measures of neonatal anthropometry, have yet to be examined.

**Objective:**

To evaluate the association between maternal caffeine intake and neonatal anthropometry, testing effect modification by fast or slow caffeine metabolism genotype.

**Design, Setting, and Participants:**

A longitudinal cohort study, the National Institute of Child Health and Human Development Fetal Growth Studies–Singletons, enrolled 2055 nonsmoking women at low risk for fetal growth abnormalities with complete information on caffeine consumption from 12 US clinical sites between 2009 and 2013. Secondary analysis was completed in 2020.

**Exposures:**

Caffeine was evaluated by both plasma concentrations of caffeine and paraxanthine and self-reported caffeinated beverage consumption measured/reported at 10-13 weeks gestation. Caffeine metabolism defined as fast or slow using genotype information from the single nucleotide variant rs762551 (CYP1A2*1F).

**Main Outcomes and Measures:**

Neonatal anthropometric measures, including birth weight, length, and head, abdominal, arm, and thigh circumferences, skin fold and fat mass measures. The β coefficients represent the change in neonatal anthropometric measure per SD change in exposure.

**Results:**

A total of 2055 participants had a mean (SD) age of 28.3 (5.5) years, mean (SD) body mass index of 23.6 (3.0), and 580 (28.2%) were Hispanic, 562 (27.4%) were White, 518 (25.2%) were Black, and 395 (19.2%) were Asian/Pacific Islander. Delivery occurred at a mean (SD) of 39.2 (1.7) gestational weeks. Compared with the first quartile of plasma caffeine level (≤28 ng/mL), neonates of women in the fourth quartile (>659 ng/mL) had lower birth weight (β = −84.3 g; 95% CI, −145.9 to −22.6 g; *P* = .04 for trend), length (β = −0.44 cm; 95% CI, −0.78 to −0.12 cm; *P* = .04 for trend), and head (β = −0.28 cm; 95% CI, −0.47 to −0.09 cm; *P* < .001 for trend), arm (β = −0.25 cm; 95% CI, −0.41 to −0.09 cm: *P* = .02 for trend), and thigh (β = −0.29 cm; 95% CI, −0.58 to −0.04 cm; *P* = .07 for trend) circumference. Similar reductions were observed for paraxanthine quartiles, and for continuous measures of caffeine and paraxanthine concentrations. Compared with women who reported drinking no caffeinated beverages, women who consumed approximately 50 mg per day (~ 1/2 cup of coffee) had neonates with lower birth weight (β = −66 g; 95% CI, −121 to −10 g), smaller arm (β = −0.17 cm; 95% CI, −0.31 to −0.02 cm) and thigh (β = −0.32 cm; 95% CI, −0.55 to −0.09 cm) circumference, and smaller anterior flank skin fold (β = −0.24 mm; 95% CI, −0.47 to −0.01 mm). Results did not differ by fast or slow caffeine metabolism genotype.

**Conclusions and Relevance:**

In this cohort study, small reductions in neonatal anthropometric measurements with increasing caffeine consumption were observed. Findings suggest that caffeine consumption during pregnancy, even at levels much lower than the recommended 200 mg per day of caffeine, are associated with decreased fetal growth.

## Key Points

**Question:**

Is maternal caffeine intake associated with neonatal anthropometry?

**Findings:**

In this cohort study of 2055 women from 12 clinical sites, measures of caffeine consumption (plasma caffeine and paraxanthine and self-reported consumption) were associated with neonatal size at birth. Increasing caffeine measures were significantly associated with lower birth weight, shorter length, and smaller head, arm, and thigh circumference.

**Meaning:**

In this study, caffeine consumption during pregnancy, even in amounts less than the recommended 200 mg per day, was associated with smaller neonatal anthropometric measurements.

## Teaser

This cohort study examines the association between maternal caffeine consumption and multiple measures of neonatal anthropometry using data from the National Institute of Child Health and Human Development Fetal Growth Studies–Singletons study.

## Introduction

Caffeine consumption during pregnancy has been an ongoing topic of debate. As of 2010, the American College of Obstetricians and Gynecologists recommends that pregnant women limit caffeine consumption to less than 200 mg per day.^1^ However, systematic reviews and meta-analyses have reported that maternal caffeine consumption, even in doses lower than 200 mg, is associated with a higher risk for low birth weight, small for gestational age (SGA), and fetal growth restriction,^2,3^ suggesting there may be no safe amount of caffeine during pregnancy. However, in 1 meta-analysis,^3^ 4 of 9 studies reported null or contrary results.^4,5,6,7^ These inconsistent associations may have been owing to the reliance of most studies on self-reported measures of caffeine intake.^2,3^ Coffee varies in its caffeine content based on preparation method, and serving size of caffeinated beverages may vary across respondents.^8^ Additionally, some studies of caffeine consumption did not control for important confounders such as smoking.^9^ Further, there are variations in individual caffeine metabolism, such that people with fast metabolism, those with a genetic variant leading to more rapid caffeine metabolism, may be at higher risk for adverse pregnancy outcomes, potentially because of higher exposure to paraxanthine, the primary metabolite in caffeine.^10,11^

To our knowledge, no studies have examined the association between caffeine intake and neonatal anthropometric measures beyond weight, length, and head circumference, and few have analyzed plasma concentrations of caffeine and its metabolites or genetic variations in the rate of metabolism associated with neonatal size.^4,10,11^ Our aim was to examine associations of caffeine consumption, based on both plasma concentrations of caffeine and its metabolites and self-reported caffeinated beverage intake, with multiple measures of neonatal anthropometry. Our secondary aim was to evaluate whether the association between caffeine consumption and neonatal anthropometry may be moderated by genetic variations in fast vs slow caffeine metabolism.

## Methods

The *Eunice Kennedy Shriver* National Institute of Child Health and Human Development (NICHD) Fetal Growth Studies–Singletons (NCT00912132) was designed to prospectively assess fetal growth in a racially/ethnically diverse cohort of pregnant women.^12^ Nonsmoking women with low-risk pregnancies, body mass index (BMI; calculated as weight in kilograms divided by height in meters squared) of 19.0-29.9, and no history of prepregnancy chronic conditions were enrolled at 12 US clinical sites between 8 and 13 weeks of gestation between 2009 and 2013.^12,13^ Secondary analysis was completed in 2020. Women were interviewed and provided blood samples. Fetal growth was tracked via ultrasonographic examinations across 6 visits. Of the 2334 women enrolled, we excluded 14 women found ineligible after enrollment, 186 with pregnancies that did not end in a live birth or with unknown birth outcomes, and 33 participants lacking information on plasma caffeine measures or self-reported caffeine consumption, leaving 2101 women and their neonates with self-reported caffeine consumption for analysis. For analyses using measured plasma caffeine and paraxanthine, we excluded 46 additional women who did not consent to have their blood samples used, leaving 2055 participants. Approval for human subjects research was obtained from the NICHD and the institutional review boards of all participating sites, and participants provided written informed consent. This study followed the Strengthening the Reporting of Observational Studies in Epidemiology (STROBE) reporting guideline for cohort studies.

### Neonatal Anthropometric Measures

Birth weight was abstracted from medical records. Neonatal anthropometric measures were obtained generally within 1 to 3 days after birth (median, 1 day; interquartile range, 1-2 days) by research staff who were trained and credentialed in a standardized manner. Length was measured from the soles of the feet to the top of the head using an electronic infant scale (SECA 416 Infantometer; SECA).^14,15,16^ Head circumference was measured by placing a tape around the head anteriorly from the forehead above the eyebrows and posteriorly at the maximum protrusion of the occiput.^17,18^ Abdominal circumference was measured by placing the measuring tape on the abdomen cephalward of the umbilicus, perpendicular to the trunk’s long mid-axis.^19,20,21,22^ Mid-upper arm circumference and mid-upper thigh circumference were measured on the right side of the body with the tape held perpendicular to the long axis and at the mid-point of the limb. All measurements were taken at least twice. A third measurement was taken if either value was higher than a prespecified technical error rate.^23,24,25^ Skin fold measures of abdominal flank, anterior thigh, subscapula, and triceps were taken using calipers. For skin fold outcomes, we excluded 136 neonates from 1 clinical site that used the wrong calipers for measurement. We calculated percent fat mass using a validated formula that combines proportions of neonatal anthropometric measures [0.39055 (neonatal examination weight, g) + 0.0453 (flank skin fold, mm) – 0.03237 (length, cm) + 0.054657].^17,26^ We examined percent fat mass as an outcome among neonates in whom the formula had been validated—those delivered at term (at least 37 weeks of gestation) or with birth weight greater than or equal to 2000 g (n = 1791). Small for gestational age was defined as birth weight below the tenth percentile for GA using the Duryea reference.^27^

### Caffeine and Paraxanthine Plasma Concentrations

Blood was collected at enrollment, processed into plasma, and stored at −80 °C. A detailed description of caffeine and paraxanthine extraction and measurement is available in the eMethods in the Supplement. Briefly, extraction was accomplished by a hybrid solid phase extraction, and quantification of caffeine was performed on a mass spectrometer (AB Sciex 5500; AB Sciex). The detection limit of caffeine through the analytical method was 0.55 ng/mL and for paraxanthine was 0.72 ng/mL and limits of quantitation were 1.85 ng/mL and 2.39 ng/mL, respectively. We assessed total methylxanthine concentrations, defined as the sum of caffeine and paraxanthine.

### Self-reported Caffeine Consumption

At enrollment participants reported whether they had consumed any caffeinated beverages in the past week (coffee, tea, soda, and energy drinks) and how many cups (8 oz) or cans or bottles (12 oz or 16 oz) consumed per day. Less than 1 serving (cup, can, or bottle) was coded as half a serving. Using USDA guidelines for average caffeine content of each beverage,^28^ we converted servings per day to milligrams per day by multiplying the number of servings by the mean caffeine content of 8 ounces of coffee (96 mg), 8 ounces of tea (48 mg), 12 ounces of soda (40 mg), or 12 ounces of energy drink (108 mg) to create a summary variable. We calculated total caffeine consumption by summing milligrams per day from all caffeine sources.

### Caffeine Metabolism and *CYP1A2* Modification

Participants were classified as having fast metabolism based on their genotype of AA and CC and slow metabolism based on their genotype of CA for the autosomal single nucleotide variant (SNV) rs762551 in the cytochrome P450 gene (*CYP1A2*), which regulates caffeine metabolism.^10,29,30,31^ DNA was extracted from stored buffy coat specimens collected at enrollment and genotyping was performed using genotyping equipment (Infinium Multi-Ethnic Global BeadChip microarray; Illumina).

### Covariates

Covariates collected at enrollment included age (years), prepregnancy BMI, race/ethnicity (non-Hispanic White, non-Hispanic Black, Hispanic, or Asian or Pacific Islander), parity (0, 1, or ≥2), married or living with partner (yes/no), educational level (<high school, high school or equivalent, some college or associate’s degree, bachelor’s degree, master’s degree or higher), insurance type (private/managed care or other), and infant sex. For all outcomes except birth weight, we adjusted for number of days elapsed between delivery and neonatal examination.

### Statistical Analysis

Descriptive analysis using χ^2^ for categorical variables and *t* tests for continuous variables were conducted to evaluate demographic differences across caffeine concentration quartiles. Pearson correlation statistics were used to compare plasma caffeine and paraxanthine with self-reported consumption. Caffeine and paraxanthine concentrations represented machine-observed values and were analyzed as quartiles (Q1, Q2, Q3, and Q4) and continuous exposures. Continuous measures were logarithm-transformed, due to skewness, and standardized after adding an appropriate positive value (eg, ln(caffeine +3)) because of negative values produced during the measurement phase. We did not substitute 0 for negative values to minimize bias associated with constraining an exposure to a lower limit.^32,33^

Using adjusted generalized linear models, we tested associations between quartiles and continuous measures of caffeine and paraxanthine concentrations and their sum relative to each neonatal outcome. To assess nonlinearity of associations for continuous exposures, we modeled log-transformed exposures as restricted cubic splines with 5 knots. The β coefficients of these models represent the change in neonatal anthropometric measure per SD change in exposure. We assessed risk of SGA using adjusted log-linear regression models.

Based on the distribution of self-reported caffeine consumption, we categorized participants as consuming no caffeine, consuming 1-50 mg per day, or consuming more than 50 mg per day, fitting models to test associations between self-reported first trimester caffeinated beverage consumption and neonatal anthropometric measures. To ensure that results were not being affected by women who consumed more than 2 cups per day (>200 mg), we performed 2 sensitivity analyses: first, we removed the individuals with the highest consumption (>200 mg/d) from analyses (n = 16), and second, we split the group who consumed more than 50 mg per day into 51 to 100 mg per day (n = 329) and more than 100 mg per day (n = 148).Because of observed race/ethnic differences in fetal growth from the NICHD cohort,^13^ in separate models, we tested for interactions between race/ethnicity and all caffeine measures for each outcome.

To test for potential moderation of caffeine metabolism *CYP1A2* genotype on the associations between caffeine consumption and neonatal anthropometry, we coded 2-way interaction terms for all caffeine exposures and rate of caffeine metabolism. All interaction models were adjusted for genetic principal components generated from multidimensional scaling analysis to account for population structure. In consideration of allele frequency differences among racial/ethnic groups, we stratified models by race/ethnicity to avoid population-stratification bias in effect estimates. Caffeine metabolism analyses included only women with genetic information to determine genotype of the *CYP1A2* gene (n = 1516).

Final models were not adjusted for plasma cotinine concentrations because it was not significantly associated with caffeine measures and did not change the results. Results were considered statistically significant at *P* < .05 in 2-tailed tests. All analyses were conducted in SAS version 9.4 (SAS Institute Inc).

## Results

A total of 2055 participants had a mean (SD) age of 28.3 (5.5) years, mean (SD) body mass index of 23.6 (3.0), and 580 (28.2%) were Hispanic, 562 (27.4%) were White, 518 (25.2%) were Black, and 395 (19.2%) were Asian/Pacific Islander. Delivery occurred at a mean (SD) of 39.2 (1.7) gestational weeks. There were no clear trends in demographic characteristics across quartiles, although women in the highest quartile (Q4) vs lowest quartile (Q1) were older (mean [SD] age: Q4, 29.5 [5.3] years vs Q1, 27.5 [5.3] years; *P* < .001) and more likely to be non-Hispanic White (No. [%]: Q4, 197 [38.4] vs Q1, 94 [18.3]; *P* < .001), parous (No. [%] ≥2: Q4, 119 [23.2] vs Q1, 54 [10.5]; *P* < .001), and married (No. [%]: Q4, 431 [84.2] vs Q1 360 [70.2]; *P* < .001) (Table 1). Plasma caffeine and paraxanthine concentrations were correlated 84% and detectable for 93% and 89% of the cohort, with median of 157 ng/mL (interquartile range, 28.3-157.2 ng/mL) and 72 ng/mL (interquartile range, 14.8-72.4 ng/mL), respectively. Pearson correlation coefficients for self-reported caffeine consumption and measured caffeine and paraxanthine were *r* = 0.33 and *r* = 0.39, respectively. Nearly half (873 of 2055 [41.6%]) of women reported consuming no caffeinated beverages in the first trimester, while 751 of 2055 women (35.7%) reported drinking at least 50 mg (approximately half a cup of coffee per day or less) and 477 of 2055 women (22.7%) reported drinking more than 50 mg per day (15.7% drank 51-100 mg/d; 6.3% drank 101-200 mg/d; 0.7% drank >200 mg/d).

**Table 1.** Sample Characteristics by Plasma Caffeine Quartiles, Fetal Growth Study–Singletons (N = 2055)

Covariate	No. (%)					P value
	All	Plasma caffeine level by quartile				
		Q1 (≤28.3 ng/mL)	Q2 (28.4-157.1 ng/mL)	Q3 (157.2-658.8 ng/mL)	Q4 (>658.8 ng/mL)	
Maternal age, y	28.3 (5.5)	27.5 (5.3)	28.3 (5.6)	27.6 (5.4)	29.5 (5.3)	<.001
Prepregnancy BMI	23.6 (3.0)	23.5 (2.9)	23.5 (3.0)	23.9 (3.2)	23.5 (2.9)	.03
Gestational age at delivery	39.2 (1.7)	39.4 (1.3)	39.2 (1.8)	39.1 (2.0)	39.2 (1.5)	.01
Race						
Non-Hispanic						<.001
White	562 (27.4)	94 (18.3)	137 (26.7)	134 (26.1)	197 (38.4)	
Black	518 (25.2)	182 (35.4)	124 (24.1)	127 (24.7)	85 (16.6)	
Hispanic	580 (28.2)	111 (21.6)	158 (30.7)	179 (34.8)	132 (25.7)	
Asian & Pacific Islander	395 (19.2)	127 (24.7)	95 (18.5)	74 (14.4)	99 (19.3)	
Infant sex						.83
Male	1058 (51.7)	257 (50.2)	268 (52.3)	262 (51.4)	271 (52.9)	
Female	988 (48.3)	255 (49.8)	244 (47.7)	248 (48.6)	241 (47.1)	
Parity						<.001
0	1007 (49.0)	298 (58.0)	276 (53.7)	242 (47.1)	191 (37.2)	
1	703 (34.2)	162 (31.5)	175 (34.1)	163 (31.7)	203 (39.6)	
≥2	345 (16.8)	54 (10.5)	63 (12.3)	109 (20.2)	119 (23.2)	
Married/living with partner	1569 (76.4)	360 (70.2)	395 (76.9)	383 (74.5)	431 (84.2)	<.001
Educational level						
<High school	207 (10.1)	56 (10.9)	45 (8.8)	60 (11.7)	46 (9.0)	.01
High school or equivalent	356 (17.3)	106 (20.6)	88 (17.1)	95 (18.5)	67 (13.1)	
Some college or associate’s degree	598 (29.1)	144 (28.0)	146 (28.4)	164 (31.9)	144 (28.1)	
Bachelor’s degree	515 (25.1)	128 (24.9)	135 (26.3)	111 (21.6)	141 (27.4)	
Master’s degree or higher	379 (18.44)	80 (15.6)	100 (19.5)	84 (16.3)	115 (22.4)	
Insurance						
Private/managed care	1359 (64.7)	331 (62.9)	349 (66.5)	313 (59.6)	366 (69.7)	.01
Other	742 (35.3)	195 (37.1)	176 (33.5)	212 (40.4)	159 (30.3)	
Caffeine metabolism^a^						
Slow	799 (52.7)	199 (54.1)	205 (55.0)	196 (49.9)	197 (50.1)	.50
Fast	717 (47.3)	169 (45.9)	168 (45.0)	197 (50.1)	183 (47.9)	
Self-reported caffeine intake, mg/d						
None	873 (41.6)	358 (41.0)	262 (30.0)	174 (19.9)	79 (9.1)	NR
1-50	751 (35.7)	132 (17.6)	198 (26.4)	227 (20.2)	194 (25.8)	
>50	477 (22.7)	36 (7.6)	65 (13.6)	124 (26.0)	252 (52.8)	

Abbreviations: BMI, body mass index (calculated as weight in kilograms divided by height in meters squared); NR, not reported.

^a^ Caffeine metabolism analyses included 1516 women with genetic information. Slow caffeine metabolism refers to CC/CA genotype, fast metabolism refers to AA genotype of the *CYP1A2* gene.

### Caffeine and Paraxanthine Plasma Concentrations

Neonatal anthropometric measures were negatively associated with quartiles of caffeine and paraxanthine concentrations with significant linear trends observed for birth weight, length, head circumference, and mid-upper arm circumference (*P* < .05 for trend) (Table 2). For caffeine, women in the highest vs lowest quartile had infants with lower birth weight (β = −84.3 g; 95% CI, −145.9 to −22.6 g; *P* = .04 for trend), shorter length (β = −0.44 cm; 95% CI, −0.78 to −0.12 cm; *P* = .04 for trend), and smaller head circumference (β = −0.28 cm; 95% CI, −0.47 to −0.09 cm; *P* < .001 for trend), mid-upper arm circumference (β = −0.25 cm; 95% CI, −0.41 to −0.09 cm: *P* = .02 for trend), and mid-upper thigh circumference (β = −0.29 cm; 95% CI, −0.58 to −0.04 cm; *P* = .07 for trend). Similar negative associations were observed for the highest vs lowest quartile of paraxanthine, with an 83.7-g reduction in birth weight (95% CI, −144.9 to −22.5 g; *P* = .01), shorter length (β = −0.45 cm; 95% CI, −0.77 to −0.12 cm; *P* = .01), and smaller head (β = −0.47 cm; 95% CI, −0.47 to −0.09 cm; *P* = .003), arm (β = -0.23 cm; 95% CI, −0.39 to −0.07 cm; *P* < .001), and thigh circumference (β = −0.31 cm; 95% CI, −0.57 to −0.05 cm; *P* = .02). Additionally, the third quartiles of paraxanthine concentrations were associated with shorter length (β = −0.38 cm; 95% CI, −0.70 to −0.06 cm; *P* = .02), and smaller head circumference (β = −0.34 cm; 95% CI, −0.53 to −0.16; *P* < .001), and mid-upper arm circumference (β = −0.19; 95% CI, −0.35 to −0.03 cm; *P* = .02) compared with the first quartile. Similar results were observed when assessing quartiles for the sum, with smaller birth weight (β = −74.4 g; 95% CI, −136.0 to −12.8 g; *P* = .02), shorter length (β = −0.46 cm; 95% CI, −0.79 to −0.13 cm; *P* = .01), and smaller head (β = −0.29 cm; 95% CI, −0.48 to −0.10 cm; *P* = .003), arm (β = −0.25 cm; 95% CI, −0.41 to −0.09 cm; *P* = 03), and thigh circumference (β = −0.28 cm; 95% CI, −0.54 to −0.02 cm; *P* = .04) (eTable 1 in the Supplement).

**Table 2.** Associations Between Caffeine and Paraxanthine Quartiles and Neonatal Anthropometric Measures, NICHD Fetal Growth Studies–Singletons (N = 2055)^a^

Outcome	Plasma caffeine level by quartile					Plasma paraxanthine level by quartile				
	β (95% CI)				*P* value for trend	β (95% CI)				*P* value for trend
	Q1 (≤28.3 ng/mL)	Q2 (28.4-157.1 ng/mL)	Q3 (157.2-658.8 ng/mL)	Q4 (>658.8 ng/mL)		Q1 (≤14.8 ng/mL)	Q2 (14.9-72.3 ng/mL)	Q3 (72.4-232.9 ng/mL)	Q4 (>232.9 ng/mL)	
Birth weight, g	1 [Reference]	−24.7 (−84.8 to 35.5)	−53.5 (−114.1 to 7.1)	−84.3 (−145.9 to −22.6)	.04	1 [Reference]	−11.1 (−71.2 to 49.0)	−56.3 (−116.9 to 4.4)	−83.7 (−144.9 to −22.5)	.03
Length, cm	1 [Reference]	−0.15 (−0.48 to 0.16)	−0.31 (−0.63 to 0.01)	−0.44 (−0.78 to −0.12)	.04	1 [Reference]	−0.16 (−0.48 to 0.16)	−0.38 (−0.70 to −0.06)	−0.45 (−0.77 to −0.12)	.03
Circumference, cm										
Head	1 [Reference]	0.01 (−0.17 to 0.20)	−0.24 (−0.43 to −0.05)	−0.28 (−0.47 to −0.09)	.001	1 [Reference]	−0.06 (−0.24 to 0.13)	−0.34 (−0.53 to −0.16)	−0.28 (−0.47 to −0.09)	<.001
Abdominal	1 [Reference]	0.03 (−0.25 to 0.31)	−0.41 (−0.42 to 0.15)	−0.26 (−0.55 to 0.03)	.17	1 [Reference]	0.03 (−0.25 to 0.31)	−0.21 (−0.49 to 0.08)	−0.21 (−0.50 to 0.08)	.20
Mid-upper arm	1 [Reference]	−0.07 (−0.22 to 0.10)	−0.13 (−0.29 to 0.03)	−0.25 (−0.41 to −0.09)	.02	1 [Reference]	−0.07 (−0.23 to 0.09)	−0.19 (−0.35 to −0.03)	−0.23 (−0.39 to −0.07)	.02
Mid-upper thigh	1 [Reference]	−0.09 (−0.34 to 0.16)	−0.28 (−0.53 to −0.02)	−0.29 (−0.58 to −0.04)	.07	1 [Reference]	−0.10 (−0.36 to 0.15)	−0.24 (−0.50 to 0.01)	−0.31 (−0.57 to −0.05)	.09
SF, mm										
Abdominal flank	1 [Reference]	−0.03 (−0.22 to 0.15)	0.01 (−0.17 to 0.20)	0.05 (−0.14 to 0.24)	.86	1 [Reference]	0.04 (−0.13 to 0.23)	0.10 (−0.09 to 0.29)	0.10 (−0.09 to 0.29)	.69
Anterior thigh	1 [Reference]	−0.05 (−0.33 to 0.17)	0.04 (−0.21 to 0.30)	−0.06 (−0.32 to 0.19)	.74	1 [Reference]	0.11 (−0.14 to 0.37)	0.03 (−0.23 to 0.28)	0.07 (−0.19 to 0.33)	.82
Subscapular	1 [Reference]	−0.12 (−0.28 to 0.05)	−0.08 (−0.25 to 0.09)	−0.01 (−0.18 to 0.16)	.45	1 [Reference]	−0.01 (−0.18 to 0.15)	−0.06 (−0.22 to 0.11)	0.05 (−0.13 to 0.22)	.69
Triceps	1 [Reference]	−0.02 (−0.21 to 0.16)	0.01 (−0.18 to 0.19)	−0.15 (−0.34 to 0.04)	.29	1 [Reference]	0.11 (−0.08 to 0.29)	−0.02 (−0.21 to 0.16)	−0.02 (−0.21 to 0.17)	.44
Fat mass, %^b^										
GA ≥37 wk	1 [Reference]	−0.01 (−0.52 to 0.51)	0.26 (−0.27 to 0.78)	−0.02 (−0.55 to 0.51)	.68	1 [Reference]	0.21 (−0.31 to 0.72)	0.31 (−0.21 to 0.84)	0.26 (−0.27 to 0.79)	.67
BW ≥2000	1 [Reference]	0.11 (−0.41 to 0.63)	0.29 (−0.24 to 0.81)	0.04 (−0.49 to 0.57)	.71	1 [Reference]	0.29 (−0.22 to 0.81)	0.32 (−0.20 to 0.84)	0.32 (−0.21 to 0.86)	.57

Abbreviations: BW, birth weight; GA, gestational age; NICHD, National Institute of Child Health and Human Development; Q, quartile; SF, skin fold.

^a^ Results of generalized linear models adjusted for maternal age, pre-pregnancy BMI, race, marital status, parity, educational level, insurance status, and infant sex. All models except for birth weight also adjusted for the number of postnatal days at measurement.

^b^ Percent fat mass is calculated using the formula: [0.39055 (neonatal examination weight, g) + 0.0453 (flank skin fold, mm) – 0.03237 (length, cm) + 0.054657].

Our findings for continuous measures were consistent with those of quartile measures, with no evidence for nonlinearity. For each SD increment increase in log caffeine, there was a decrease in birth weight (β = −26.3 g; 95% CI, −47.4 to −5.0 g; *P* = .004), length (β = −0.14 cm; 95% CI, −0.26 to −0.03 cm; *P* = .02), and smaller head (β = −0.09 cm; 95% CI, −0.16 to −0.02 cm; *P* = .01), and arm circumference (β = −0.06 cm; 95% CI, −0.12 to −0.01 cm; *P* = .04). For log paraxanthine, there was a decrease in birth weight (β = −24.5 g; 95% CI, −45.5 to −3.4 g; *P* = .01), length (β = −0.18 cm; 95% CI, −0.29 to −0.06 cm; *P* = .003), and smaller head (β = −0.13 cm; 95% CI, −0.20 to −0.06 cm; *P* < .001), arm (β = −0.08 cm; 95% CI, −0.13 to −0.02 cm; *P* = .01), and thigh circumference (β = −0.10 cm; 95% CI, −0.19 to −0.01 cm; *P* = .04). For each SD increment increase in the log of the sum, there was a decrease in birth weight (β = −25.9 g; 95% CI, −47.1 to −4.8 g; *P* = .004), length (β = −0.18 cm; 95% CI, −0.30 to −0.06 cm; *P* = .003), and smaller head (β = −0.13 cm; 95% CI, −0.20 to −0.06 cm; *P* < .001), arm (β = −0.08 cm; 95% CI, −0.14 to −0.02 cm; *P* = .01), and thigh circumference (β = −0.10 cm; 95% CI, −0.19 to −0.01 cm; *P* = .04) (Table 3). Risk of SGA was elevated in the fourth quartile of caffeine (adjusted relative risk = 1.26; 95% CI, 0.83-1.91) and paraxanthine (adjusted relative risk = 1.31; 95% CI, 0.85-2.00) (Table 4). There were no significant interactions between race and any measure of caffeine consumption (eTable 2 in the Supplement).

**Table 3.** Associations Between Continuous Caffeine and Paraxanthine Plasma Concentrations and Neonatal Anthropometric Measures, NICHD Fetal Growth Studies–Singletons (N = 2055)^a^

Outcome	Continuous plasma biomarkers (log-transformed and standardized), β (95% CI)		
	Caffeine	Paraxanthine	Caffeine + paraxanthine sum
Biomarker, mean (SD), ng/mL	604.8 (1120.4)	167.5 (240.1)	772.3 (1327.9)
Birth weight, g	−26.25 (−47.44 to −5.04)	−24.48 (−45.52 to −3.44)	−25.94 (−47.09 to −4.79)
Length, cm	−0.14 (−0.26 to −0.03)	−0.18 (−0.29 to −0.06)	−0.18 (−0.30 to −0.06)
Circumference, cm			
Head	−0.09 (−0.16 to −0.02)	−0.13 (−0.20 to −0.06)	−0.13 (−0.20 to −0.06)
Abdominal	−0.02 (−0.12 to 0.08)	−0.09 (−0.20 to 0.01)	−0.10 (−0.20 to 0.00)
Mid-upper arm	−0.06 (−0.12 to −0.01)	−0.08 (−0.13 to −0.02)	−0.08 (−0.14 to −0.02)
Mid-upper thigh	−0.05 (−0.14 to 0.04)	−0.10 (−0.19 to −0.01)	−0.10 (−0.19 to −0.01)
SF, mm			
Abdominal flank	0.04 (−0.03 to 0.10)	0.05 (−0.01 to 0.12)	0.05 (−0.02 to 0.12)
Anterior thigh	0.00 (−0.09 to 0.09)	0.02 (−0.08 to 0.11)	0.02 (−0.07 to 0.11)
Subscapular	−0.01 (−0.07 to 0.05)	0.01 (−0.05 to 0.07)	0.01 (−0.05 to 0.07)
Triceps	−0.06 (−0.12 to 0.01)	−0.03 (−0.10 to 0.04)	−0.04 (−0.11 to 0.03)
Fat mass, %^^b^^			
GA ≥37 wk	0.10 (−0.09 to 0.28)	0.13 (−0.05 to 0.32)	0.11 (−0.08 to 0.29)
BW ≥2000	0.11 (−0.07 to 0.30)	0.15 (−0.04 to 0.34)	0.12 (−0.07 to 0.31)

Abbreviations: BW, birth weight; GA, gestational age; NICHD, National Institute of Child Health and Human Development; SF, skin fold.

^a^ Results of generalized linear models adjusted for maternal age, pre-pregnancy BMI, race, marital status, parity, educational level, insurance status, and infant sex. All models except for birth weight also adjusted for the number of postnatal days at measurement. β can be interpreted as the unit change in anthropometric measure per SD increase in biomarker concentration.

^b^ Percent fat mass is calculated using the formula: [0.39055 (neonatal examination weight, g) + 0.0453 (flank skin fold, mm) – 0.03237 (length, cm) + 0.054657].

**Table 4.** The aRR of Small for Gestational Age for Plasma Caffeine and Paraxanthine Quartiles, NICHD Fetal Growth Studies–Singletons (N = 2055)^a^

Variable	aRR (95% CI)			
	Q1	Q2	Q3	Q4
Plasma caffeine				
SGA	1 [Reference]	0.84 (0.54-1.30)	0.82 (0.53-1.27)	1.26 (0.83-1.91)
Plasma paraxanthine				
SGA	1 [Reference]	1.01 (0.66-1.57)	1.03 (0.67-1.59)	1.31 (0.85-2.00)

Abbreviations: aRR, adjusted relative risk; NICHD, National Institute of Child Health and Human Development; Q, quartile; SGA, small for gestational age.

^a^ SGA represents birth weight less than the tenth percentile for gestational age, based on the Duryea reference^27^; number of SGA neonates = 180 (9.6%).

### Self-reported Consumption

Coffee and soda were the primary sources of caffeine consumption, with 35% of participants (736 of 2101) consuming coffee and 41% (870 of 2101) consuming soda. Analyses were conducted based on reports at the enrollment visit, but caffeinated beverage intake was reported across visits and remained consistent for 98% of women in the second and third trimesters. Compared with women who reported no caffeinated beverage intake, women who reported drinking at least 50 mg per day had neonates with smaller subscapular skin folds (β = −0.14 mm; 95% CI, −0.27 to −0.01 mm**)** and women who reported drinking more than 50 mg per day had neonates with lower birth weight (β = −66 g; 95% CI, −121 to −10 g) and smaller mid-upper arm circumference (β = −0.17 cm; 95% CI, −0.31 to −0.02 cm), mid-upper thigh circumference (β = −0.32 cm; 95% CI, −0.55 to −0.09 cm), and anterior thigh skin fold (β = −0.24 mm; −0.47 to −0.01 mm) (Table 5). Results were robust in sensitivity analysis.

**Table 5.** Associations Between Self-reported Caffeine Consumption and Various Neonatal Outcomes (N = 2101)^a^^,^^b^

Outcome	Caffeine consumption		
	None (n = 873 [41.55%])	1-50 mg/d (n = 751 [35.74%])^^c^^	>50 mg/d (n = 477 [22.70%])
Birth weight, g	1 [Reference]	−21.72 (−69.89 to 26.46)	−65.93 (−121.39 to −10.47)
Length, cm	1 [Reference]	−0.03 (−0.28 to 0.22)	−0.21 (−0.51 to 0.08)
Circumference, cm			
Head	1 [Reference]	0.09 (−0.06 to 0.24)	−0.11 (−0.28 to 0.07)
Abdominal	1 [Reference]	−0.02 (−0.24 to 0.21)	−0.20 (−0.46 to 0.05)
Mid-upper arm	1 [Reference]	−0.09 (−0.22 to 0.04)	−0.17 (−0.31 to −0.02)
Mid-upper thigh	1 [Reference]	−0.20 (−0.40 to 0.003)	−0.32 (−0.55 to −0.09)
SF, mm			
Abdominal flank	1 [Reference]	−0.09 (−0.24 to 0.06)	0.12 (−0.05 to 0.29)
Anterior thigh	1 [Reference]	−0.17 (−0.37 to 0.03)	−0.24 (−0.47 to −0.01)
Subscapular	1 [Reference]	−0.14 (−0.27 to −0.01)	0.02 (−0.13 to 0.17)
Triceps	1 [Reference]	−0.12 (−0.27 to 0.02)	−0.02 (−0.19 to 0.15)
% Fat mass^d^			
GA ≥37 wk	1 [Reference]	−0.01 (−0.42 to 0.41)	0.03 (−0.44 to 0.51)
BW ≥2000	1 [Reference]	0.08 (−0.34 to 0.49)	0.08 (−0.39 to 0.56)

Abbreviations: BW, birth weight; BMI, body mass index (calculated as weight in kilograms divided by height in meters squared); GA, gestational age; SF, skin fold.

^a^ Sample includes 46 women who were excluded from blood sample analyses.

^b^ Results of generalized linear models adjusted for maternal age, prepregnancy BMI, race, marital status, parity, educational level, insurance status, and infant sex. All models except for birth weight also adjusted for the number of postnatal days at measurement.

^c^ Caffeine contents for common beverages includes 96 mg for 8 oz of coffee, 48 mg for 8 oz of tea, 40 mg for 12 oz of soda, and 108 mg for 12 oz of energy drink.

^d^ Percent fat mass is calculated using the formula: [0.39055 (neonatal examination weight, g) + 0.0453 (flank skin fold, mm) – 0.03237 (length, cm) + 0.054657].

### Caffeine Metabolism

In testing interactions between metabolism rate and caffeine or paraxanthine quartiles, there were no significant interactions for any neonatal anthropometric measure indicating that the association between caffeine concentrations and neonatal anthropometry did not vary by rate of caffeine metabolism. Results were consistent for self-reported measures.

## Discussion

In this cohort study of pregnant women with low caffeine consumption, even small increases in plasma caffeine concentrations and its major metabolite paraxanthine, were associated with lower birth weight, finding that smaller size was manifested by shorter length, and smaller head, arm and thigh circumferences at birth. The decreases in bone and muscle measures, but not skin folds and fat mass, may indicate decreases in lean tissue as caffeine consumption increases. Results were consistent with self-reported caffeine consumption, in which consumption of at least 50 mg (approximately half a cup of coffee) per day was associated with lower birth weight and smaller neonatal anthropometric measurements, even when excluding individuals who consumed higher amounts (>200 mg). Associations between caffeine and neonatal anthropometric measures did not vary by fast or slow caffeine metabolism.

Our findings of decreased birth weight and length associated with both plasma caffeine and paraxanthine concentrations and caffeinated beverage intake are consistent with meta-analyses that have reported a dose-response association between self-reported maternal caffeine consumption and the risk of low birth weight, SGA, and fetal growth restriction,^2,3,34^ although associations with measured caffeine have not previously been established. Pooled statistics in these analyses demonstrated a graded risk in low birth weight that increases with each additional cup of coffee (100 mg) consumed per day, suggesting that even low amounts of caffeine consumption during pregnancy are associated with smaller offspring birth size. Similarly, we observed no threshold effect for caffeine consumption, as shown by associations between caffeine biomarkers and anthropometric measurements, and the finding that even low consumption of caffeinated beverages was associated with less lean tissue, which may have long-term implications for cardiometabolic risk.^35^ To our knowledge, few studies have explored caffeine consumption in association with neonatal anthropometric measures beyond birth weight and length.^10,36,37^ Our results are consistent with 2 studies that additionally explored head circumference,^10,36^ although our sample was larger, and our detailed measures specifically characterized changes in lean and fat tissue. In addition, we observed these associations in a cohort of pregnant women with low mean caffeine consumption (36 mg/d). Our findings are in contrast to null associations with neonatal weight, length, and head circumference observed in another study conducted in a low-consumption sample,^37^ however, that study included only 100 women, possibly lacking power to detect an association.

Other studies assessing caffeinated beverage intake instead of measured caffeine and paraxanthine concentrations have suggested increased risk of negative growth outcomes such as SGA and fetal growth restriction only after consumption of 200 to 300 mg per day.^4,38^ Consistent with this finding, we observed higher risk for SGA in the fourth quartile of measured caffeine and paraxanthine concentrations, although results were not significant, likely because of low overall consumption in the sample. However, SGA is an extreme of birth weight, and does not describe incremental changes that may signal a negative association between caffeine and neonatal size. When evaluating outcomes continuously, a 2018 study noted linear decreases in birth weight, length, and head circumference with increasing self-reported caffeine consumption, as observed in our study.^36^ We were unable to directly calculate plasma caffeine or paraxanthine concentrations to the amount of caffeine consumed. However, it is estimated that among pregnant women in their first trimester, consuming 55 mg of caffeine translates to a mean blood caffeine concentration of 1859 ng/mL an hour after consumption.^39^ In the context of our study, when comparing the fourth with the first caffeine quartiles, a 630 ng/mL increase in caffeine concentration translated to an 84 g reduction in birth weight and a 0.44 cm decrease in length. Thus, our results indicate that even small increases in caffeine consumption in the first trimester may translate to reductions in neonatal anthropometric measures, and our findings were robust in multiple analyses.

The long-term implications of our findings are unclear, considering the relatively small estimates we observed. Caffeine metabolism slows throughout pregnancy.^39^ Because the fetus lacks *CYP1A2* enzymes for metabolism, caffeine and paraxanthine accumulate in fetal tissues.^40^ Caffeine is hypothesized to alter fetal growth via disruption of neuroendocrine processes that cause uteroplacental vasoconstriction, hinder organ development, and permanently alter the stress response.^40^ In the long term, these disruptions may put offspring at higher risk for rapid weight gain after birth, childhood obesity, and chronic disease.^41,42^ Even low maternal caffeine intake (>50 mg/d) is associated with higher risk of excess growth in infancy and overweight in early childhood and altered fat deposition that may put children of caffeine consumers at higher risk of later cardiometabolic disease.^41,43^

Although evidence supports high interindividual variation in the rate of caffeine metabolism,^10,11,44^ we did not observe a modifying effect of caffeine metabolism. However, this null finding may, in part, be owing to the low level of consumption in our sample. To date, only 1 study has examined caffeine metabolism genotypes in the context of pregnancy, finding that differences in associations between caffeine and neonatal anthropometry only differed by metabolism rate among high-consumption groups (≥300 mg/d).^10^ Additionally, our sample was racially/ethnically diverse, which necessitated further stratification by race, limiting the power to detect a small effect. Although we used a validated SNV to define fast and slow metabolism, there are likely multiple genes involved in caffeine metabolism, and in a low-consumption sample, a single SNV may not be a sensitive indicator of metabolism alone.

### Strengths and Limitations

A major strength of our study was the ability to investigate caffeine intake from multiple measures including plasma caffeine and paraxanthine concentrations, self-reported caffeine consumption, and genetic information on caffeine metabolism. Unlike previous studies which relied mostly on coffee consumption,^38^ our self-reported measure included caffeinated coffee, tea, soda, and energy drinks. Another strength is the numerous, rigorously collected anthropometric measures, which allowed us to investigate associations with neonatal lean and fat measures. In addition, by limiting our sample to nonsmokers without chronic disease, we reduced unmeasured confounding in our analyses.

This study has limitations. Similar to other studies assessing first trimester consumption,^45^ there was low correlation between self-reported caffeinated beverage intake and plasma caffeine and paraxanthine in our sample, possibly because of variability in caffeine amounts from beverage intake,^8^ differential rates of metabolism, and lack of information on timing of last consumption. By using biomarker data, we overcame many of these limitations and recorded caffeine exposure from consuming certain foods, such as chocolate and decaffeinated beverages, which may contain small amounts of caffeine. We measured plasma caffeine and paraxanthine once in pregnancy, although it should be noted that self-reported caffeine remained constant for 98% of the sample in the second and third trimesters. This finding is consistent with other caffeine studies that found stable reported consumption across trimesters,^4,5^ and little variability in results based on timing of exposure.^4^ Thus, measuring caffeine in the first trimester may be a good proxy of consumption throughout pregnancy, but evaluation of caffeine biomarker changes across trimesters may be warranted in future studies.

## Conclusions

In this cohort study, we observed small reductions in neonatal anthropometric measurements with increasing caffeine consumption. Our results suggest that caffeine consumption during pregnancy, even at levels much lower than the recommended 200 mg per day of caffeine^1^ may be associated with decreased fetal growth.

## Notes

Supplement. **eMethods.** Description of Caffeine and Paraxanthine Quantification **eTable 1.** Associations Between Caffeine-Paraxanthine ∑ Quartiles and Neonatal Anthropometric Measures, NICHD Fetal Growth Studies-Singletons, n = 2.055 **eTable 2.** Characteristics of the Sample Included for Genetic Analyses, by Race (n = 1,516) **eReference**
