# Supplementary material for: Association Between Maternal Caffeine Consumption and Metabolism and Neonatal Anthropometry: A Secondary Analysis of the NICHD Fetal Growth Studies–Singletons
Source: JAMA Netw Open. 2021 Mar 25;4(3):e213238. doi: 10.1001/jamanetworkopen.2021.3238 (PMC7994948; doi:10.1001/jamanetworkopen.2021.3238)
Supplement: Supplement. — eMethods. Description of Caffeine and Paraxanthine Quantification eTable 1. Associations Between Caffeine-Paraxanthine ∑ Quartiles and Neonatal Anthropometric Measures, NICHD Fetal Growth Studies-Singletons, n = 2.055 eTable 2. Characteristics of the Sample Included for Genetic Analyses, by Race (n = 1,516) eReference [file jamanetwopen-e213238-s001.pdf]

Supplemental Online Content

Gleason JL, Tekola-Ayele F, Sundaram R, et al. Association between maternal caffeine consumption and metabolism and neonatal anthropometry: a secondary analysis of the NICHD Fetal Growth Studies–Singletons. *JAMA Netw Open*. 2021;4(3):e213238. doi:10.1001/jamanetworkopen.2021.3238

eMethods, Description of Caffeine and Paraxanthine Quantification

eTable 1. Associations Between Caffeine-Paraxanthine Σ Quartiles and Neonatal Anthropometric Measures, NICHD Fetal Growth Studies-Singletons, n=2,055

eTable 2. Characteristics of the Sample Included for Genetic Analyses, by Race (n=1,516)

eReference

This supplemental material has been provided by the authors to give readers additional information about their work.

© 2021 Gleason JL et al. *JAMA Network Open*.

eMethods: Description of caffeine and paraxanthine quantification

Extraction of caffeine and paraxanthine was accomplished by a hybrid solid phase extraction (SPE), similar to that reported elsewhere.[1] Briefly, plasma samples (250 µL) were transferred into a 15-mL polypropylene tube, spiked with 5 ng each of isotopically labeled internal standards, and mixed with 100 µL of 10% ammonium hydroxide (*v*/*v*). The samples were mixed and incubated at room temperature for 30 minutes then mixed with 810 µL of methanol containing 1% ammonium formate (*w/v*) and vortexed. The samples were centrifuged at 4000 rpm for 10 min and the supernatants were pipette transferred and passed through a hybrid SPE cartridge (Phospholipid, 1 mL, 30 mg, Supelco; Waltham, MA, USA) that was pre-conditioned with 1% ammonium formate in methanol (1 mL). The eluates were collected into autosampler vials for instrumental analysis. The chromatographic separation of caffeine and paraxanthine was accomplished using a Waters Acquity I-Class UPLC system (Milford, MA, USA) connected with an Acquity UPLC BEH C18 column (50 × 2.1 mm, 1.7 µm, Waters; Milford, MA, USA). Identification and quantification of caffeine was performed on an ABSCIEX 5500 (Applied Biosystems; Foster City, CA, USA) with a sample injection volume of 3 µL. The mobile phase comprised of methanol (A) and HPLC grade water containing 0.05% ammonium hydroxide (*v*/*v*) and 0.05% formic acid (*v*/*v*) (B). The detection limit of caffeine and paraxanthine through the analytical method was 0.55 and 0.72 ng/mL, respectively, and the limits of quantitation were 1.85 and 2.39 ng/mL, respectively. The average recovery of caffeine through the SRMs was 110±11%. The recoveries of caffeine and paraxanthine spiked into commercial serum was 100±5.8% and 92.2±7.8%. Caffeine and paraxanthine were present in procedural blanks at 0.26 and 0.27 ng/mL, respectively.

© 2021 Gleason JL et al. *JAMA Network Open*.

eTable 1. Associations between caffeine-paraxanthine Σ quartiles and neonatal anthropometric measures, NICHD Fetal Growth Studies-Singletons, n=2,055

	Plasma Caffeine-Paraxanthine Σ Quartile				
	Q1 (≤45.7 ng/mL)	Q2 (45.8 – 237.9 ng/mL)	Q3 (238.0 – 914.0 ng/mL)	Q4 (>914.0 ng/mL)	***P* trend**
Birth Weight, g	[Ref]	-11.32 (-71.55, 48.90)	**-62.76 (-123.47, -2.06)**	**-74.41 (-136.02, -12.80)**	0.04
Length, cm	[Ref]	-0.21 (-0.53, 0.11)	**-0.36 (-0.68, -0.04)**	**-0.46 (-0.79, -0.13)**	0.03
Head circumference, cm	[Ref]	-0.02 (-0.21, 0.17)	**-0.27 (-0.46, -0.09)**	**-0.29 (-0.48, -0.10)**	0.001
Abdominal Circ., cm	[Ref]	-0.02 (-0.30, 0.26)	-0.18 (-0.46, 0.10)	-0.27 (-0.56, 0.02)	0.19
Mid-upper arm Circ., cm	[Ref]	-0.06 (-0.22, 0.10)	-0.09 (-0.26, 0.07)	**-0.25 (-0.41, -0.09)**	0.02
Mid-upper thigh Circ., cm	[Ref]	-0.10 (-0.36, 0.15)	**-0.29 (-0.55, -0.03)**	**-0.28 (-0.54, -0.02)**	0.08
Abdominal Flank SF, mm	[Ref]	-0.02 (-0.20, 0.17)	0.02 (-0.16, 0.21)	0.08 (-0.11, 0.27)	0.76
Anterior thigh SF, mm	[Ref]	0.01 (-0.24, 0.26)	0.06 (-0.20, 0.32)	0.004 (-0.25, 0.26)	0.96
Subscapular SF, mm	[Ref]	-0.08 (-024, 0.09)	-0.03 (-0.20, 0.14)	0.03 (-0.14, 0.20)	0.63
Triceps SF, mm	[Ref]	0.02 (-0.17, 0.20)	0.04 (-0.15, 0.23)	-0.09 (-0.28, 0.10)	0.52
Percent fat mass (GA≥37wk)	[Ref]	0.22 (-0.30, 0.73)	0.34 (-0.19, 0.86)	0.17 (-0.36, 0.70)	0.65
Percent fat mass (BW≥2000)	[Ref]	0.30 (-0.22, 0.82)	0.36 (-0.17, 0.89)	0.22 (-0.31, 0.75)	0.56

Note: Results of generalized linear models adjusted for maternal age, pre-pregnancy BMI, race, marital status, parity, educational attainment, insurance status, and infant sex. All models except for birthweight also adjusted for the number of postnatal days at measurement.
*Percent fat mass is calculated using the formula: [0.39055 (neonatal exam weight) + 0.0453 (flank skin fold) – 0.03237 (length) + 0.054657]

© 2021 Gleason JL et al. *JAMA Network Open*.

eTable 2. Characteristics of the sample included for genetic analyses, by race (n=1,516)

	All	White	Black	Hispanic	Asian/PI
Total *n*	1516	471	424	418	203
Mean caffeine, ng/mL (SD)	598.1 (1111.1)	830.7 (1296.8)	414.9 (950.6)	603.2 (1113.1)	542.9 (990.5)
Mean paraxanthine, ng/mL (SD)	166.3 (238.8)	233.1 (293.6)	127.6 (191.9)	147.4 (190.7)	163.34 (254.1)
Caffeine Quartile					
1st	368 (24.3%)	73 (15.5%)	149 (35.1%)	76 (18.2%)	70 (34.5%)
2nd	373 (24.6%)	111 (23.6%)	101 (23.8%)	117 (28.0%)	44 (21.7%)
3rd	393 (25.9%)	116 (24.6%)	104 (24.5%)	131 (31.3%)	42 (20.7%)
4th	382 (25.2%)	171 (36.3%)	70 (16.5%)	94 (22.5%)	47 (23.2%)
Caffeine consumption					
None	609 (40.2%)	180(38.2%)	179 (42.2%)	170 (40.7%)	80 (39.4%)
Up to 50 mg/day	557 (36.7%)	172 (36.5%)	166 (39.2%)	137 (32.8%)	82 (40.4%)
>50mg/day	350 (23.1%)	119 (25.3%)	79 (18.6%)	111 (26.6%)	41 (20.2%)
CYP1A2 genotype					
CC	142 (9.4%)	41 (8.7%)	52 (12.3%)	29 (6.9%)	20 (9.9%)
CA	657 (43.3%)	192 (40.8%)	204 (48.1%)	174 (41.6%)	87 (42.9%)
AA	717 (47.3%)	238 (50.5%)	168 (39.6%)	215 (51.4%)	96 (47.3%)
Caffeine metabolism					
Slow	799 (52.7%)	233 (49.5%)	256 (60.4%)	203 (48.6%)	107 (52.7%)
Fast	717 (47.3%)	238 (50.5%)	168 (39.6%)	215 (51.4%)	96 (47.3%)

*Slow caffeine metabolism refers to CC/CA genotype, fast metabolism refers to AA genotype

eReference

1. Honda M, Robinson M, Kannan K. A rapid method for the analysis of perfluorinated alkyl substances in serum by hybrid solid-phase extraction. *Environ. Chem.* 2018;15(2):92-99.

© 2021 Gleason JL et al. *JAMA Network Open*.
